# A stable lithiated silicon–chalcogen battery via synergetic chemical coupling between silicon and selenium

**DOI:** 10.1038/ncomms13888

**Published:** 2017-01-05

**Authors:** KwangSup Eom, Jung Tae Lee, Martin Oschatz, Feixiang Wu, Stefan Kaskel, Gleb Yushin, Thomas F. Fuller

**Affiliations:** 1 Center for Innovative Fuel Cell and Battery Technologies, School of Chemical and Biomolecular Engineering, Georgia Institute of Technology, 311 Ferst Drive, NW, Atlanta, Georgia 30332, USA; 2 School of Materials Science and Engineering, Gwangju Institute of Science Technology (GIST), 123 Cheomdangwagi-ro, Buk-gu, Gwangju 61005, South Korea; 3 School of Materials Science and Engineering, Georgia Institute of Technology, Atlanta, Georgia 30332, USA; 4 Department of Inorganic Chemistry, Dresden University of Technology, Bergstrasse 66, 01069 Dresden, Germany

## Abstract

Li-ion batteries dominate portable energy storage due to their exceptional power and energy characteristics. Yet, various consumer devices and electric vehicles demand higher specific energy and power with longer cycle life. Here we report a full-cell battery that contains a lithiated Si/graphene anode paired with a selenium disulfide (SeS_2_) cathode with high capacity and long-term stability. Selenium, which dissolves from the SeS_2_ cathode, was found to become a component of the anode solid electrolyte interphase (SEI), leading to a significant increase of the SEI conductivity and stability. Moreover, the replacement of lithium metal anode impedes unwanted side reactions between the dissolved intermediate products from the SeS_2_ cathode and lithium metal and eliminates lithium dendrite formation. As a result, the capacity retention of the lithiated silicon/graphene—SeS_2_ full cell is 81% after 1,500 cycles at 268 mA g_SeS2_^−1^. The achieved cathode capacity is 403 mAh g_SeS2_^−1^ (1,209 mAh cm_SeS2_^−3^).

## Abstract (web summary)

Lithium-based batteries employing silicon anodes and sulfur cathodes are promising for combining low cost and high capacity, but have been limited in terms of cycling stability. Here authors present cycling and characterization data supporting beneficial synergies between a selenium disulfide cathode and a silicon anode.

Lithium-ion batteries (LiBs) have been successfully commercialized and are widely used in portable electronic devices, electric and hybrid-electric vehicles, aerospace applications and even grid-scale facilities. The continued, rapid technological advancement of these systems requires batteries with higher specific power/energy, longer cycle life and competitive costs^1^,^2^,^3^. However, the present state-of-the-art LiBs cannot keep pace with the emerging needs; hence, it is essential to search for alternative battery materials and chemistries^4^,^5^.

Transition metal oxides (cathode or positive electrode) and graphite (anode or negative electrode) are the predominant active materials used in commercial LiBs. Their material production, processing and electrochemical behaviour are relatively well understood. These two materials have relatively low gravimetric capacities and improvements have nearly plateaued. Therefore, alternative materials are being investigated. Among the many candidates, silicon for the anode and sulfur for the cathode are of great interest due to their natural abundance, low intrinsic cost and high energy-storage capabilities^6^,^7^. Silicon (Si)^7^, for example, has a theoretical specific capacity of 4,200 mAh g^−1^, about ten times that of graphite, and sulfur (S)^8^,^9^ has a theoretical capacity of 1,672 mAh g^−1^. The successful combination of these two materials would be a breakthrough in the battery research field. Specifically, it is desirable for a high capacity anode to be matched with a high capacity cathode. If a high capacity Si anode is paired with a commercially available metal transition oxide, for example, nickel manganese cobalt oxide (NMC) cathode, the NMC cathode would be much thicker compared with the Si anode. As diffusion time increases with the square of diffusion length, battery performance, especially rate capability, dramatically diminishes.

That being said, combining S and Si to produce a high-capacity battery has been exceedingly challenging. In fact, even adoption of a single material is problematic due to the continuous degradation (capacity fade) during long-term LiB cycling. For instance, Si expands ∼300% during lithiation, resulting in its pulverization and a large irreversible capacity loss^10^. As an electrical conductor and a buffer material, graphene can increase the rate capability and cycle life of the Si electrode as used in the form of Si/graphene composites^6^,^11^,^12^. Nevertheless, continuous reduction in the utilization of the active material due to the formation of fragile solid electrolyte interphase (SEI) and its endless breakdown/growth is the biggest barrier to a long-term (more than 1,000 cycles) use of Si-graphene electrode^13^. A high-quality SEI with high conductivity and mechanical durability during long-term cycling is necessary.

The key challenges in electrochemistry of Li/S battery systems are as follows: (1) the loss of active material due to the dissolution of high-order lithium polysulfides (Li_2_S_*x*_, *x*≥4) in the electrolyte and (2) the persistence of unwanted reactions between Li metal and dissolved S species, and (3) dendrite formation on the Li metal anode. The first problem can be partially avoided by adopting porous carbon, which contains the sulfur inside nanopores based on overlapping of adsorption potentials^9^,^14^,^15^,^16^,^17^. The second problem could be largely circumvented by employing the appropriate electrolyte additive(s) to form the desirable SEI^18^. However, these strategies are still not satisfactory, and further improvements are required. A more fundamental approach would be to find an alternative to Li metal for the anode.

Recently, several studies reported a full cell employing both S cathodes and Si anodes^19^,^20^,^21^. They demonstrated an interesting concept, although the capacity fade was rapid. These cells showed limited cyclability—none of these studies demonstrated more than several tens of cycles. We believe that the dissolved S species lowers the stability of Si as well as itself (S); hence, in this study we propose to add a good SEI-forming element, to the cathode. Selenium (Se) is a promising cathode material^22^. It has high volumetric (∼3,260 mAh cm^−3^ in a Li-free state) and gravimetric (678 mAh g^−1^) capacities, similar to S. In contrast, the electrical conductivity of Se is several orders of magnitude higher than that of S. The electrical conductivity of Se thin film (0.1∼0.5 μm)^23^ is 10^−6^∼10^−8^ S cm^−1^, whereas that of solid sulfur^24^ is 10^−15^∼10^−30^ S cm^−1^. In our previous study^14^, we have shown that Se forms a stable SEI on Li due to its much higher ionic conductivity. The chalcogens react with each other to form interchalcogen compounds; hence, formulating multiple chalcogen materials as battery materials and property control (that is, magnitude of capacity, electrical conductivity, cost and so on) will be facile. The use of Se_*x*_S_*y*_ would be beneficial to increase gravimetric capacity^25^ and form higher-quality SEI.

The plateau voltage of the Si-S/Se_*x*_S_*y*_ systems (1.7∼2.0 V) are about 1.5∼2.1 V lower than that of traditional lithium metal oxide cathodes (3.7∼4.3 V). However, due to higher specific capacity of both materials (Si: 4,200 mA hg^−1^, S: 1,672 mAh g^−1^), it is notable that the Si-S battery system offers up to ∼2 times higher gravimetric (specific) energy density (up to ∼700 Wh kg^−1^) than the present graphite-lithium metal oxide systems (<350 Wh kg^−1^ in commercial cells).

For our proof-of-concept studies presented here, we choose selenium disulfide (SeS_2_)/carbide-derived carbon (CDC) for the cathode and Si/graphene for the anode to construct a full-cell battery system. To the best of our knowledge, these complementary materials have not been reported, and for the first time we demonstrate attractive performance of the lithiated Si/Graphene−SeS_2_/CDC full cell. We further propose a mechanism for the origin of this performance. Selenium, dissolved from the SeS_2_ cathode, becomes a component of the SEI on the Si/graphene anode, resulting in the significant increase of its conductivity and stability. The replacement of Li metal anode by the lithiated silicon/graphene retards dissolution of intermediate products from the SeS_2_ cathode and formation of Li dendrite in the anode.

## Results

### Si/graphene anode and SeS_2_/CDC cathode

**Figure 1:**
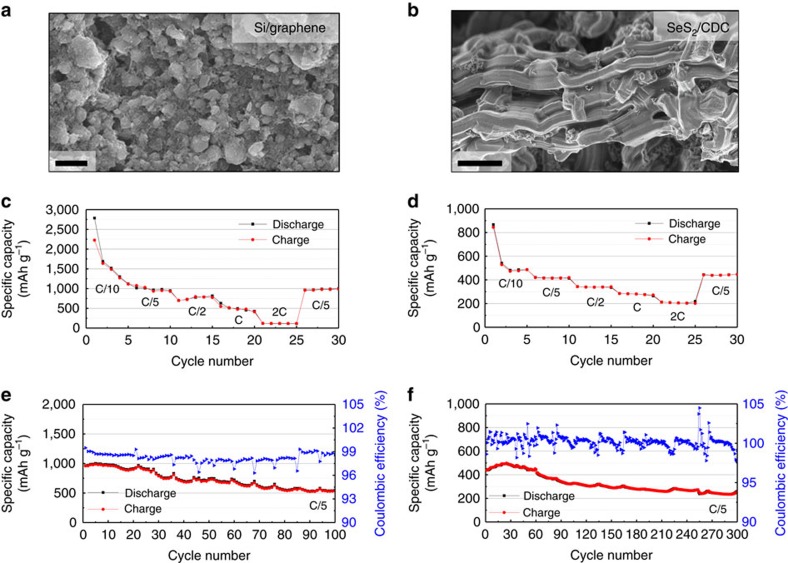
Surface morphology and electrochemical performance (half cell) of Si/graphene and SeS_2_/CDC electrodes. High-resolution SEM surface images (× 10,000) of (**a**) Si/graphene and (**b**) SeS_2_/CDC electrodes. The half-cell tests of Si/graphene for anode and SeS_2_/CDC for cathode were performed between 0.05–1.50 V and 1.0–2.85 V, respectively, at 20 °C. The rate capability of both half-cells employing (**c**) Li–Si/graphene and (**d**) Li–SeS_2_/CDC at the lithiation/delithiation rates between C/10 and 2C. The CR and CE of each half cells of (**e**) Li–Si/graphene and (**f**) Li–SeS_2_/CDC at C/5. Scale bars, 2 μm (**a**,**b**).

Figure 1 displays the surface morphologies and electrochemical LiB performances of the lithiated Si/graphene and SeS_2_/CDC electrodes. The lithiated Si/graphene electrode has agglomerates of spherical Si particles (300 nm∼4 μm) and micrometre-sized plate-like multi-layered graphene (Fig. 1a). The size of a SeS_2_/CDC nanocomposite is ∼15 μm in length and ∼5 μm in diameter. One particle is composed of multiple small nanoporous rods and SeS_2_ is embedded in these pores. No SeS_2_ agglomerates are observed on the surface of the CDC particle, indicating that most of SeS_2_ exist in the pores. The electrochemical LiB performance of the electrodes were characterized in half cells using Li metal as a reference/counter electrode in diethyl carbonate (DEC) based electrolyte with 1 M lithium hexafluorophosphate salt at 20 °C. The lithiated Si/graphene anode and SeS_2_/CDC cathode were cycled between 0.05–1.50 and 1.0–2.85 V, respectively. The rate-capability tests (Fig. 1c,d) were performed at lithiation/delithiation rates between C/10 and 2C, and the cycling tests were conducted at C/5 (1C is based on its theoretical capacity.). The capacity of the lithiated Si/graphene significantly decreased with increasing C-rate from C/10 to 2C; specifically, the average capacity at 2C was only 14.5% of that at C/5. On the other hand, the SeS_2_/CDC electrode exhibited relatively good rate capability: at 2C 49.5% of the C/5 capacity was preserved. The good rate capability of SeS_2_-CDC originated mainly from the unique structure of CDC. The CDC is composed of straight mesopores (2−8 nm) and small micropores (< 2 nm). Not only is ion transport greatly improved through aligned pores, but the electron access significantly increases, because SeS_2_ is trapped in the small pores and is in contact with conductive carbon framework^14^,^26^. In addition, the smaller feature size of SeS_2_ results in the better rate capability. Figure 1e,f show the capacity retention (CR) and coulombic efficiency (CE) of the lithiated Si/graphene and SeS_2_/CDC electrode with LiB cycling. The Si/graphene and SeS_2_/CDC electrodes exhibited the CR decrement of 56.4% (963.6→543.8 mAh g^−1^) for 100 cycles and 58.4% (444.1→259.2 mAh g^−1^) after 300 cycles. The large decrements could be proven from a result that the CEs of the both electrodes are mostly below 100%. Therefore, from the half-cell results it appears that both electrodes would be impractical—as a guide the US Department of Energy's target for an electric vehicle (EV) battery is 80% CR after 1,000 cycles^27^. Previous studies have identified the main causes of degradation of the Si/graphene half-cell and SeS_2_ half-cell (including S and Se). In Si systems, capacity fade has been ascribed to the continuous Si pulverization and concomitant SEI growth leading to loss of cyclable Li and conductivity decay^7^,^28^. For the S (Se) electrode, the dissolution of intermediate S (Se) compound and its unwanted reactions between Li metal has been found responsible for reducing CR^29^,^30^.

### Design of full cells and their electrochemical performance

**Figure 2:**
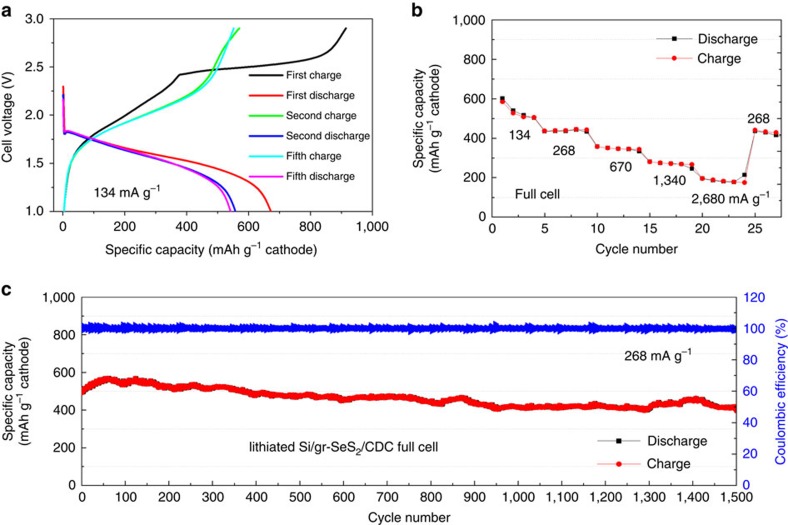
Electrochemical performances of the newly designed full cell employing lithiated Si/graphene anode and SeS_2_/CDC cathode. The full cell was tested between 1.0 and 2.85 V at the charge/discharge rates from 134 to 2,680 mA g_SeS2_^−1^ at 20 °C. (**a**) The charge/discharge curves during the first five formation cycles. (**b**) The rate capability with an increase in charge/discharge rate from 134 to 2,680 mA g_SeS2_^−1^ and then a decrease to 134 mA g_SeS2_^−1^. (**c**) The CR and CE at 268 mA g_SeS2_^−1^ for 1,500 cycles.

**Figure 3:**
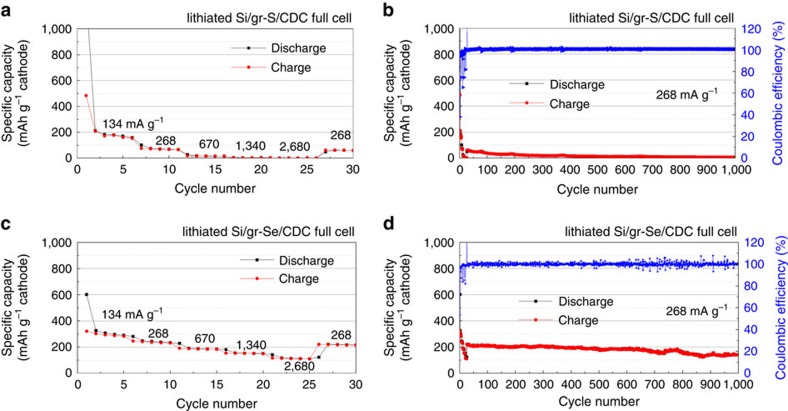
Electrochemical performance of half and full cells employing S/CDC and Se/CDC electrodes for comparative study. The CR and CE of (**a**) half cell and (**b**) full cell employing Si/graphene−S/CDC electrode, and (**c**) half cell and (**d**) full cell employing Si/graphene−Se/CDC electrode. All the test conditions are same with those for lithiated Si/graphene–SeS_2_/CDC full cells; all the cells were tested between 1.0 and 2.85 V at the charge/discharge rates from 134 to 2,680 mA g_SeS2_^−1^ at 20 °C.

The initial charge/discharge processes of the full cell employing the lithiated Si/graphene anode and SeS_2_/CDC cathode are shown in Fig. 2a and the rate capability of the cell between C/10 and 2C is displayed in Fig. 2b. The first lithiation capacity was 915 mAh g_SeS2_^−1^, but decreased to 672 mAh g_SeS2_^−1^ after the subsequent delithiation; that is, the first cycle CE is 73.5%. It is due to additional formation of the SEI^13^ and the loss of cyclable lithium during the first formation cycle^10^. During the next four cycles the capacity slightly decreased and at the fifth cycle the charge and discharge capacity was measured to be 552 and 541 mAh g_SeS2_^−1^ (98.1% CE). After the fifth cycle, the cell no longer exhibited such obtrusive capacity fade, as shown in Fig. 2b,c. As seen in the rate-capability test of Fig. 2b, the capacity decreased with an increase in the charge/discharge rate. At the highest specific current of 2,680 mA g_SeS2_^−1^, which is 20 times higher than that of initial cycle, the specific capacity was stable between 175 and 195 mAh g_SeS2_^−1^. When reducing the current to 268 mA g_SeS2_^−1^ after the rate-capability test, the capacity was fully recovered to 441 mAh g_SeS2_^−1^, which is the original level at the same rate, implying that there was no degradation during initial rate capability testing. Figure 2c shows the cyclability at 268 mA g_SeS2_^−1^ during the subsequent 1,500 cycles. The curve shows a slight decrease from 498 to 403 mAh g_SeS2_^−1^ over 1,500 cycles. Hence, the rate of capacity fade is calculated to be 63 μAh g_SeS2_^−1^cycle^−1^ and the CR is 81% over 1,500 cycles with an average CE of 99.99%. In the magnified CEs of Supplementary Fig. 1, it is shown that the all values are staying near 100%. The large decrement of 2% is sometimes shown, but recovered soon in the subsequent cycle, which means the CE decrement is temporary during full charge–discharge process. These results indicate that the full-cell can be cycled practically at least 1,500 times. Moreover, the capacity based on the cathode material mass after 1,500 cycles is 3∼5 times higher than that in the recently reported full cells, such as graphite or silicon anode—lithium cobalt oxide (LCO), NMC or lithium nickel cobalt aluminum oxide (NCA) cathode^11^,^13^,^31^,^32^. It is notable that the performance of the lithiated Si/graphene (Si-graphene)–SeS_2_/CDC full cell is remarkable. The full cells employing S/CDC and Se/CDC electrodes were assembled for the comparative study with the SeS_2_/CDC full cell. In all cases, lithiated Si/graphene was used for the anode and the cell performances are presented in Fig. 3. The S/CDC full cell showed poor rate capability and cyclability. In contrast, the Se/CDC full cell showed the rate capability as good as SeS_2_/CDC full cell; however, the specific capacity is about two times smaller than the SeS_2_/CDC full cell (222 and 142 mAh g_SeS2_^−1^ during 1,000 cycles). It is noteworthy that the half-cell of S/CDC also showed better rate capability and then that the capacity could be fully recovered to original level (∼410 mAh g_SeS2_^−1^) at 268 mA g_SeS2_^−1^ (Supplementary Fig. 2). This change in capacity implies the S/CDC half cell behaves differently from the full cell. Typically a full cell has poorer rate capability and CR than a half cell^11^,^13^. For example, Supplementary Fig. 3 shows the LiB performance of a lithium titanate (LTO) half cell and the full cell that has a voltage window similar to SeS_2_, S and Se electrodes. The cell voltage of the full cell is ∼0.2 V lower than half cell, because the Si electrode has ∼0.2 V higher reversible potential for lithiation/delithiation than a Li anode. The LTO itself is known to have good rate capability (Supplementary Fig. 3); however, when it is paired with Si/graphene anode, the capacity dramatically decreased 4.5 times from C/5 to C/2. LTO also demonstrated high rate capability up to 5C when it was used with LiNi_0.5_Mn_1.5_O_4_ as a full cell^33^. Hence, it is concluded that the rate-limiting factor originated from Si anode is likely to be due to the development of resistive SEI formation. In contrast, the lithiated Si/graphene−SeS_2_/CDC full cell has higher CR and rate capability than both individual half cells (Li−Si/graphene and Li−SeS_2_/CDC). These results clearly indicate that there is synergistic effect between Si and SeS_2_.

### *Post mortem* micro-structure and surface morphology

For material characterization of electrodes, all the tested cells were opened in an Ar-filled glove box. Before analysis, all the electrodes were gently washed to remove Li salts and stored in containers filled with Ar to prevent air exposure. When the lithiated Si/graphene–SeS_2_/CDC coin-type full cell was opened after 1,500 cycles, it was observed that there was no obvious, macroscopic degradation, such as electrolyte dry out, contamination of electrolyte and electrodes, and fracture and separation of electrodes (Supplementary Fig. 4).

Supplementary Fig. 5 shows the high-resolution scanning electron microscopy (SEM) images of the electrodes after 1,000 cycles. When compared with the pristine images of each electrode (Fig. 1a,b), the cycled lithiated Si/graphene−SeS_2_ and lithiated Si/graphene−Se full cells kept their original morphologies. In contrast, the lithiated Si/graphene−S full cell exhibited surface destruction of both electrodes. Specifically, the Si/graphene anode has some agglomerates on the surface and the surface of S/CDC cathode became rough with agglomerated spherical particles mainly due to the deposition of low-order polysulfides. Supplementary Fig. 6a,b show the X-ray photoelectron spectroscopy (XPS) depth profile of the cycled S/CDC and SeS_2_ /CDC electrodes. With increasing etch time (going towards the bulk), the S content increased and remained saturated at a constant ratio (bulk content), implying that S is slightly dissolved from surface of both CDC electrodes and a passivating layer containing mainly carbon is formed on the surface of CDC (Supplementary Fig. 7). The XPS analysis (Supplementary Fig. 8) of the cycled lithiated Si/graphene anodes indicates that there is elemental S on the anode surface of the full cell employing SeS_2_/CDC cathode, which showed the best CR. This suggests that both full cells exhibit S dissolution from the cathode and deposition on the anode, and thus should have a similar impact on the cell degradation. Considering that the depth profiles of S in S/CDC and SeS_2_/CDC are almost identical, and that the stability of SeS_2_/CDC full cell is excellent, both S/CDC and SeS_2_/CDC cathodes should be equally electrochemically stable. Indeed, we observed the S/CDC is stable when it is paired with Li metal (Supplementary Fig. 2a). Hence, we can conclude that the fast degradation of the S full cell might be ascribed primarily to the lithiated Si/graphene anode.

**Figure 4:**
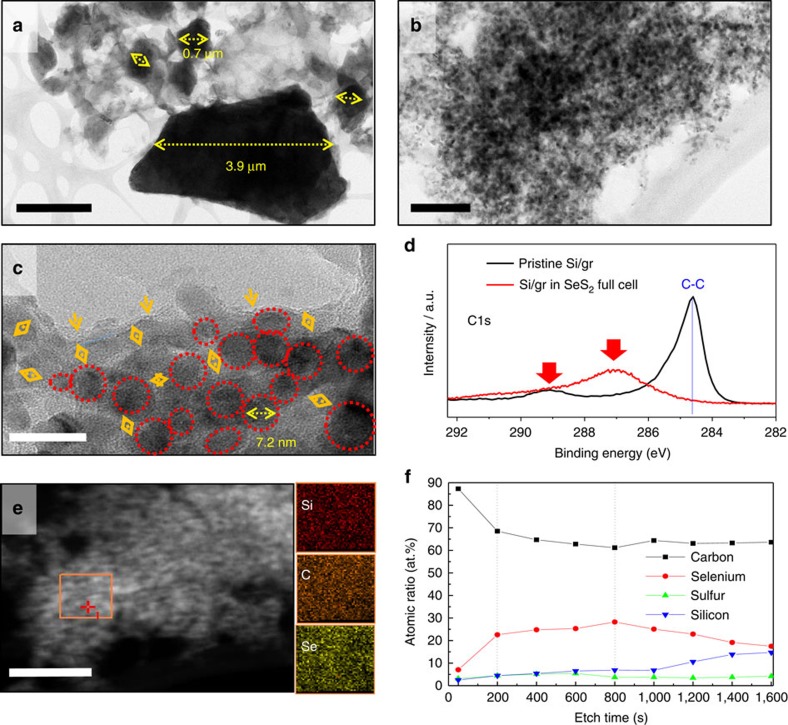
High-resolution TEM (HR-TEM)/STEM images and XPS depth profile of lithiated Si/graphene-SeS2/CDC full cell after 1,000 cycles. HR-TEM bright-field images of (**a**) pristine and (**b**) cycled lithiated Si/graphene anode. (**c**) Highly magnified TEM image of **b**. (**d**) XPS surface analysis for C1s of the cycled lithiated Si/graphene electrode. (**e**) STEM/atomic mapping of cycled lithiated Si/graphene anode for silicon (Si), carbon (C) and selenium (Se). (**f**) XPS surface depth profile of the cycled lithiated Si/graphene anode. Scale bars, 2 μm (**a**), 100 nm (**b**), 20 nm (**c**) and 200 nm (**e**).

In the nano-sized scale, the lithiated Si/graphene electrode showed significant changes in morphology. Figure 4a,b show transmission electron microscope (TEM) bright-field images of Si/graphene electrode before and after 1,000 cycles. Pristine Si particles are well mixed with the graphene plates and range between 100 nm and 4 μm, as shown in Fig. 4a. However, after 1,000 cycles the particle size greatly decreased to nanoscale of 5–10 nm as shown in Fig. 4b,c. It might be attributed to the continuous pulverization of Si particles due to the repeated expansion and contraction during LiB cycles^13^. In terms of pulverization, loss of active materials induces capacity fade, whereas a decrease in particle size to nanoscale improves the rate capability and hence retains higher capacity even at faster C-rate. The SEI is formed along the surface of the active material particles and may occupy the gaps between the pulverized particles.

In Fig. 4c, the 2–5 nm grey-coloured layers (indicated by orange arrows) covering the dark spots (red circles) are thought to be SEI formed between nano-sized Si particles. From the XPS surface analysis of C1s (Fig. 4d), it is confirmed that SEI compounds, which are composed of C=O (287 eV) and O–C=O (289 eV)^34^, fully covered the surface of the lithiated Si/graphene electrode (within XPS surface detection level) and, therefore, C–C bonding of graphene was not detected. As the SEI is composed of oxides and is an insulator, its formation may thus electrochemically isolate individual Si nanoparticles that form during Si pulverization and greatly decrease the electrical conductivity of the Si/graphene electrode and reduce its capacity. Many previous studies have focused on the formation of a thin and stable SEI using electrolyte additive(s) or surface-treatment techniques and incremental improvements have been shown^35^,^36^,^37^. However, the superiority of this approach is the demonstration of the ability to spontaneously form a higher quality SEI *in situ* by properly designing a full cell.

In the scanning TEM/energy dispersive X-ray spectroscopy (EDS) analysis of Fig. 4e, Se-containing species were found to be uniformly distributed on pulverized Si nanoparticles. We hypothesize that such species may electrically connect individual Si nanoparticles at the inner layer of the anode SEI and improve their electrochemical stability during subsequent cycling by forming an ionically conductive buffer layer. From the XPS depth profile of Fig. 4f, it is notable that the layer has different Se composition profile as a function of each time and the middle section of SEI layer has a large amount of atomic Se of 25–30 at.%. The dQ/dV curves (Supplementary Fig. 9b) for the first five cycles show that the relatively high peaks are detected between 1.49 and 1.76 V. These broad peaks originate from the low order polychalcogenides (Li_2_S(or Se)_*x*_ where *x*<4) considering the cell voltage of the full cell is ∼0.2 V lower than that of the half cell^25^,^38^. Subsequently, during the first charge, the abnormal and high peaks are observed between 2.42 and 2.52 V due to the SEI formation on anode^13^. Then, it is considered that Se could be contained, while forming the anode SEI, because there are enough sources of Se in the form of Li_2_Se_*x*_ (*x*≥4) in the electrolyte. A recent study reported that if a chalcogen element is confined in a small space, it can directly convert from elemental chalcogen to low-order polychalcogenide showing only one plateau^39^. In our study, only the formation discharge cycle showed very small peak at 2.29 V, which is attributed to the formation of highly soluble high-order polychalcogenides (Li_2_S(or Se)_*x*_ where *x*≥4) decreasing the CE. The SeS_2_ located in larger pores of CDC formed high-order polychalcogenides and dissolved into the electrolyte. After that, only SeS_2_ retained in the small pores of CDC did not dissolved into the electrolyte and electrochemically cycled.

### Electrochemical impedance spectroscopy during full-cell cycling

**Figure 5:**
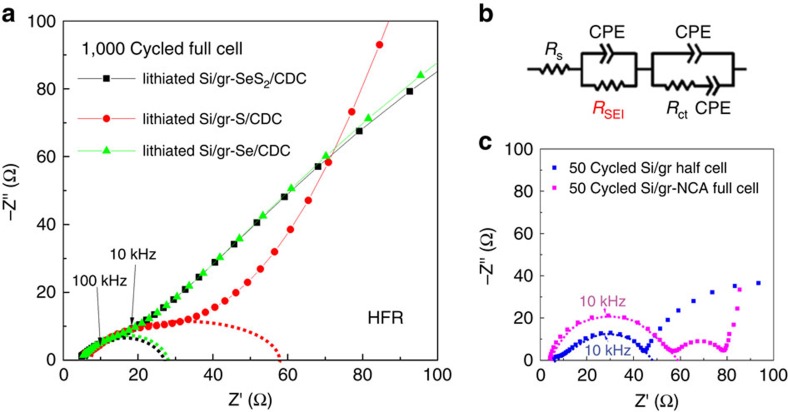
EIS of the three full cells after 1,000 cycles. (**a**) EIS curves of the three cycled full cells of lithiated Si/graphene–SeS_2_/CDC, lithiated Si/graphene–S/CDC and lithiated Si/graphene–Se/CDC electrodes. (**b**) Expected equivalent circuit of the full cells, where *R*_SEI_ is related to the diameter of HFR semicircle in **a**. (**c**) EIS curves of 50 cycled Si/graphene half cell and lithiated Si/graphene–SeS_2_/CDC full cell. The *R*_SEI_ calculated from fitting in HFR are 21.0, 21.4, 52.6, 44.2 and 52.6 Ω for lithiated Si/graphene–SeS_2_/CDC, lithiated Si/graphene–S/CDC and lithiated Si/graphene–Se/CDC full cells, Si/graphene half cell and Si/graphene–lithium nickel cobalt aluminum oxide (NCA) full cell, respectively.

Electrochemical impedance spectroscopy (EIS) is a powerful tool to investigate the kinetics of reactions and diffusion processes occurring in the electrodes, surface films and electrolyte^40^,^41^,^42^. A typical Nyquist plot for the full-cell LiB consists of a high-frequency *Z*_im_-intercept, first high-frequency semi-circle, the second middle-frequency semi-circle and a low-frequency curve. These features are roughly attributed to the resistances of electrolyte, the resistance of the SEI films (mostly on negative electrode), the charge-transfer resistance and diffusion, respectively^43^. Figure 5a displays the EIS curves of full cells of lithiated Si/graphene–S/CDC, lithiated Si/graphene–SeS_2_/CDC and lithiated Si/graphene–Se/CDC electrodes after 1,000 cycles. Our main interest is on high-frequency resistance (HFR) region, determined by the resistance of electrolyte and SEI. Specifically, the semi-circle presenting in HFR region is directly related to conductivity of SEI, which determines the quality of SEI for high LiB performance^35^,^37^,^40^. The diameter of the HFR semicircle is equal to the SEI resistance including electrical and ionic resistances (mostly by ionic conductivity)^40^,^41^. The SEI resistances of the three full cells calculated by fitting are 21.0, 21.4 and 52.6 Ω for the lithiated Si/graphene–Se/CDC, lithiated Si/graphene–SeS_2_/CDC and lithiated Si/graphene–S/CDC full cells, respectively, which can support that the full cells employing Se-containing cathodes has a high-quality SEI with high conductivity. The values are also smaller than those of the 50 cycled Si/graphene half cell (52.6 Ω) and Si/graphene–NCA full cell (44.2 Ω), indicating that Se-containing SEI can keep higher ionic conductivity even after a long-term cycle (1,000 cycles) than generally formed SEI on Si anode without Se. In particular, from the changes in the EIS curves of the lithiated Si/graphene–SeS_2_/CDC full cell with cycle number (Supplementary Fig. 10), it was found that despite increasing the cycle number from 100 to 1,500, EIS curves in the HFR range are unchanged.

## Discussion

**Figure 6:**
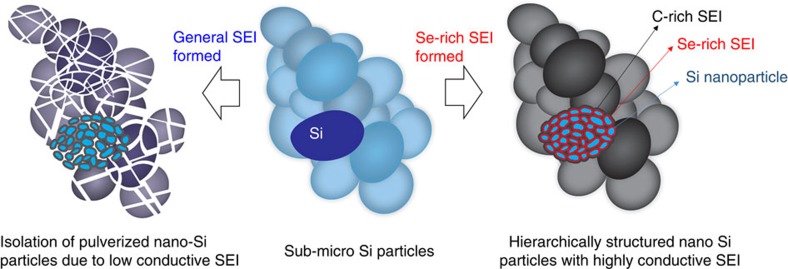
Schematic of the in-situ formation of hierarchically structured nano-Si particles with Se-containing SEI layer. During the LiB cycles, micro-sized Si particles are pulverized to nano-sizes. Then, the formation of general low-conductive SEI leads to continuous growth of SEI on the pulverized surface and then induces a large increase in resistance and loss of the cyclable lithium ions, resulting in rapid capacity decay (isolation of active material). In contrast, when the cathode material has Se or Se compounds (SeS_*x*_), the dissolved Se ions from cathode becomes an essential component of SEI (hierarchical layer structure).The highly conductive SEI can keep the Li ion/electron transfer between electrolyte and active materials fast and hence impede an unnecessary SEI growth on the surface and the isolation of the pulverized Si particles into the non-electrochemically active area.

On the basis of the results presented, the mechanism for synergetic effect from coupling between silicon anode and selenium sulfide cathode, which demonstrated high capacity and long-term stability for LiB, can be proposed as shown in the schemes of Fig. 6. During the LiB cycles, micro-sized Si particles are pulverized to nano-sizes due to the repeated expansion and contraction. Then, the formation of general poorly conductive SEI and continuous growth of such an SEI on the pulverized surface induces a significant increase in resistance, loss of cyclable Li ions and electrochemical isolation of individual Si nanoparticles, resulting in a rapid capacity fade. When the cathode material has a Se or Se compounds (SeS_*x*_), however, dissolved Se ions from the cathode become an essential component of the SEI. It is thought that its portion close to Si is electrically conductive, thus connecting individual Si nanoparticles. In addition, Se contributes to making the SEI highly ionically conductive, more mechanically robust and more resistive to solvent penetration. A highly conductive SEI ensures that the Li ion/electron transfer reactions between the electrolyte and active materials are fast and hence impede an unnecessary SEI growth on the surface and the separation the pulverized Si particles to the not-electrochemically active area. Further, the replacement of Li metal by lithiated Si/graphene as an anode can also improve the stability of LiB by impeding the unwanted side reactions between dissolved intermediate products and Li metal, and Li dendrite formation that plague Li/S or Se cells.

In summary, we demonstrated remarkable CR of the lithiated Si/graphene–SeS_2_ full cell, <20% degradation after 1,500 cycles. More important, our studies suggest that the improvements in the electrochemical stability of high-capacity Si anode may be realized by using cathodes containing Se. We expect that our findings will provide new avenues for the improvements in the Si SEI stability by incorporating Se (and possibly similar materials) species exhibiting similar properties or behaviour.

## Methods

### Si-graphene anode preparation

A commercially available Si-graphene composite was used for the anode. The active material was composed of nano Si particles and nano-sized plate-like multi-layer graphene (XG sciences, xGnP). To make the slurry of adhesive and conductive anode material, the nano-sized active material was combined with polyacrylic acid (PAA) binder (Sigma Aldrich, *M*_v_: 450,000 g mol^−1^), carbon black conductive agent and conductive additive of micro-sized multi-layer granular graphene with very high conductivity of 10^7^ S m^−1^, and propyleneglycol monomethyl ether solvent (Sigma Aldrich). After mixing the slurry by sonication and stirring with magnetic bar in a jar, the slurry was coated on a 10 μm Cu foil using a doctor blade. The coating on Cu foil was dried for 1 h at a room temperature and then in a vacuum oven at 80 °C overnight. The loading active of active material was between 1.64 and 1.66 mg cm^−2^.

### CDC-wrapped S/Se/SeS_2_ cathode preparation

The selenium sulfide (SeS_2_) melted at 120 °C and was absorbed into the CDC pores based on capillary action. Both inner and outer surfaces of the carbon were coated during the infiltration process. Any extra SeS_2_ that coated the outside of the pores, which could easily dissolve and lower the sulfur utilization/the active mass during cycling, was evaporated at 200 °C. From the gas sorption (Supplementary Fig. 11) and thermo-gravimetric analysis (Supplementary Fig. 12) analyses, it was confirmed that most of pores were successfully filled with SeS_2_ and stronger bonding between SeS_2_ and CDC was formed within nano-pores of the SeS_2_/CDC, respectively. The SeS_2_/CDC powders and PAA (Sigma Aldrich) binder were mixed in ethanol/water solution to prepare the slurry for casting the electrode. Pure black (Superior Graphite) and purified exfoliated graphite (Superior Graphite) were used as conductive additives, and the mass ratio was 75:15:5:5, respectively, SeS_2_/CDC, PAA binder, pure black and purified exfoliated graphite. The slurry was stirred at room temperature for 12 h and cast on aluminum foil. After drying overnight at room temperature under vacuum, the electrodes were used.

### Li-ion cell assembly

The coin cells were assembled with 1 M lithium hexafluorophosphate in ethylene carbonate:diethyl carbonate (1:1, v-v) as electrolyte, Celgard 2325 separator, silicon/graphene composite anode and S/Se/SeS_2_ CDC cathodes in a glove box filled with Ar gas at room temperature. All of coin cells are 2,032 type and each cell has a 1 mm stainless-steel spacer and one spring (Belleville washers) on cathode side. One hundred and fifty microlitres of electrolyte was added to 2,032 coin cells with active materials of 1 mg cm^−2^ in both anode and cathode. The cells were equilibrated for 24 h before operation. For Li addition to the full-cells, the Si/graphene anode was lithiated to 0.005 V at C/10 rate before assembly of the full cell and then the lithiated Si/graphene was used. To see the effects of both the anode and cathode on the capacity fade of the full cell during cycling, the capacity ratio of anode:cathode was designed to be close to 1:1. Using the measured gravimetric capacity (mAh g^−1^) during initial stable cycle (1–10 cycles at C/5) for the half cells employing of Si/graphene anode, SeS_2_/CDC, S/CDC and Se/CDC cathodes, the loading weights (g) were determined for full-cell design. The thickness of all the electrodes range between 25 and 33 μm.

### Electrochemical measurements

To characterize the electrochemical properties of Li-ion batteries employing various fabricated electrodes, capacity–voltage (*C*–*V*) and EIS test were performed. All cycling tests were conducted at room temperature (∼20 °C) using a battery cycler (Arbin). The half cell of Si-graphene anode was charged to 1.5 V and discharged to 0.01 V (1 h rest time between charge and discharge) at various rates from 134 to 2,680 mA g_SeS2_^−1^ for capacity-fade testing. Although the half cells of the Se/S/SeS_2_ CDC cathodes and full cells employing the electrodes were charged to 2.85 V and discharged to 1.0 V at various rates from 134 mA g_SeS2_^−1^ to 2,680 mA g_SeS2_^−1^. EIS tests of the full cells were conducted at both a nearly full charged state using a potentiostat (Autolab). The frequency was scanned from 1 MHz∼0.01 Hz using a 5 mV amplitude perturbation. The values for SEI resistances of individual components in high-resistance frequency were determined with a fitting program (Gamry Echem Analyst).

### Material characterization

For material characterization of electrodes, the cells were opened in an Ar-filled glove box and washed gently for 2 min in extra pure dimethyl carbonate (C_3_H_6_O_3_, Aldrich) to remove Li salts fully and the samples were stored in containers filled with Ar before analysis to prevent any contact with air. To observe the surface morphology and phases of nano-sized Si particles and SEI, high-resolution TEM equipped with EDS (FEI Tecnai F30, 300 kV) was used. From the EDS line scanning in scanning TEM mode, atomic mapping images were obtained. To examine the chemical composition of electrode surface, X-ray photoelectron spectroscopy (Thermo K-Alpha XPS) analysis was performed. The vacuum transfer module was used to prevent air exposure. Using etching by the Ar cluster ion beam, XPS depth profiles were obtained. In addition, high-resolution SEM (Hitachi SU8000) and SEM/EDS (Zeiss Leo 1530) were used to observe the micro-surface morphology and atomic compositions of materials. N_2_ sorption and thermo-gravimetric analysis were also used to measure surface area and to see thermal properties of CDC and SeS_2_/CDC materials for cathode, respectively.

### Data availability

The authors declare that the data supporting the findings of this study are available within the article and its Supplementary Information files. All other relevant data supporting the findings of this study are available on request.

## Additional information

**How to cite this article:** Eom, K. *et al*. A stable lithiated silicon–chalcogen battery via synergetic chemical coupling between silicon and selenium. *Nat. Commun.* **8,** 13888 doi: 10.1038/ncomms13888 (2017).

**Publisher's note**: Springer Nature remains neutral with regard to jurisdictional claims in published maps and institutional affiliations.

## Supplementary Material

Supplementary Information Supplementary Figures
