# Supplementary material for: A stable lithiated silicon–chalcogen battery via synergetic chemical coupling between silicon and selenium
Source: Nat Commun. 2017 Jan 5;8:13888. doi: 10.1038/ncomms13888 (PMC5227063; doi:10.1038/ncomms13888)
Supplement: Supplementary Information — Supplementary Figures [file ncomms13888-s1.pdf]

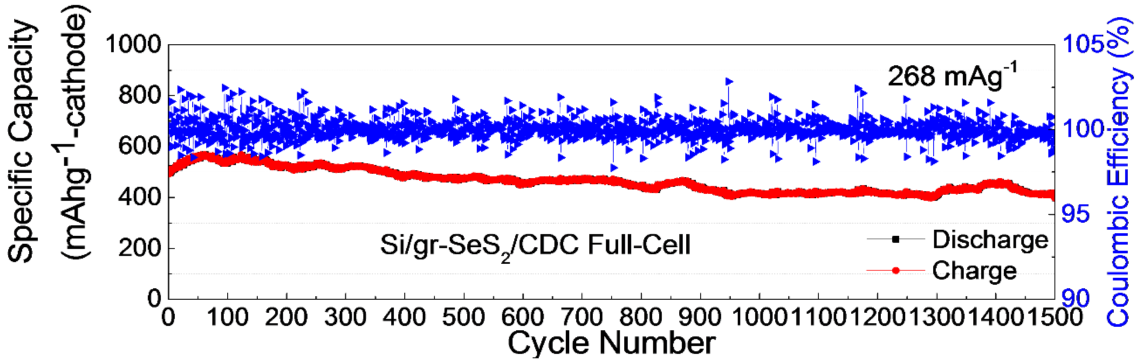

Supplementary Figure 1 │ Coulombic efficiency of Si/Gr – SeS_2 full-cell. The highly magnified coulombic efficiency of **Fig. 2c**. The test was conducted at 268 mA g_{SeS2}^{-1} for 1500 cycles.

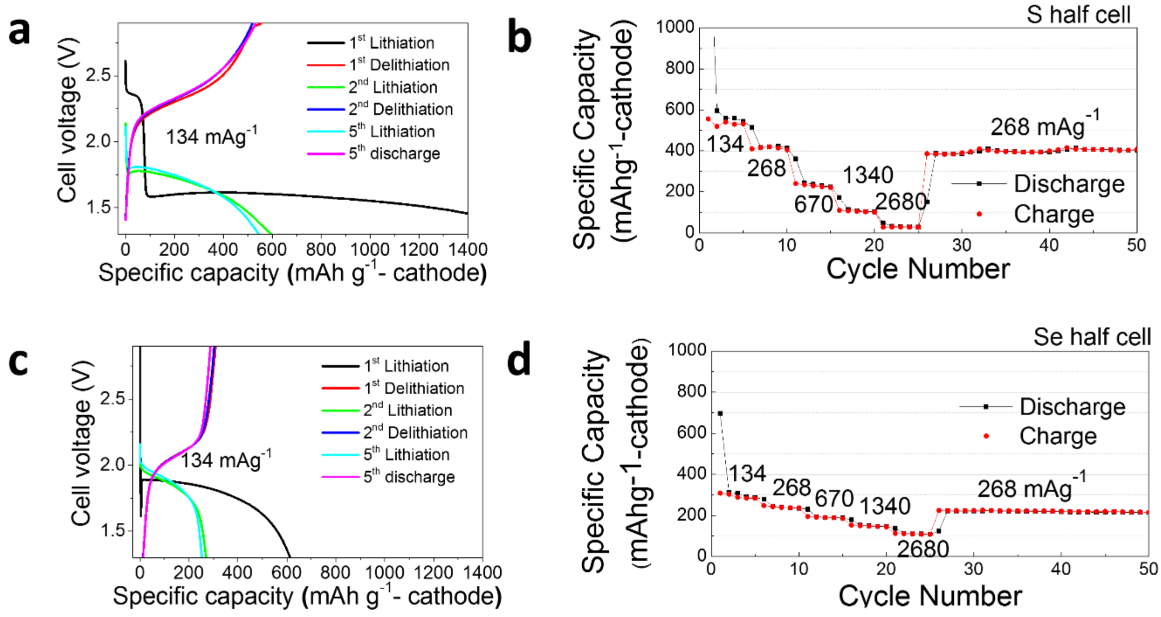

Supplementary Figure 2 │ Electrochemical performance of half-cells employing S/CDC and Se/CDC electrodes for comparative study. The charge/discharge curves during the first five formation cycles and rate-capability tests of half-cells employing (a and b) S/CDC electrode and (c and d) Se/CDC electrode. All the test conditions are the same as those for Si/graphene – SeS_2/CDC full cells; all the cells were cycled between 1.30 and 2.90 V at the charge/discharge rates from 134 to 2,680 mAg^{-1} at 20 ^{o}C.

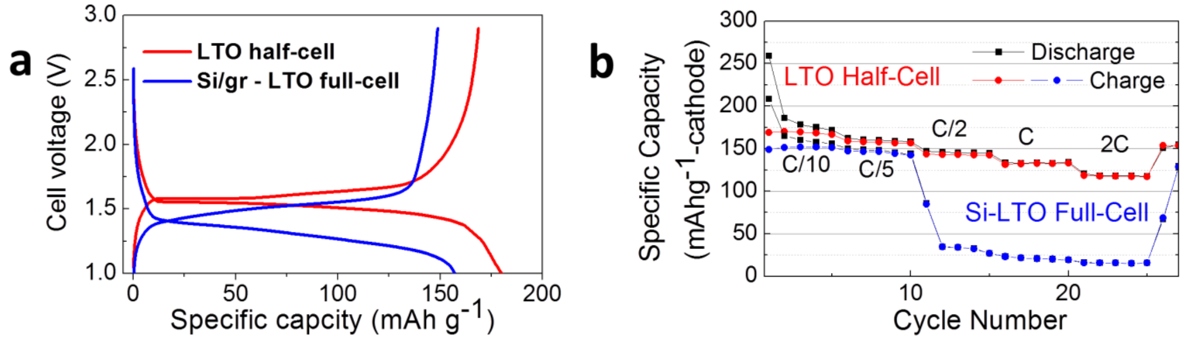

Supplementary Figure 3 │ Electrochemical performance of the LTO half-cell and Si/Gr – LTO full-cell. (a) The rate-capability tests of the two cells from C/10 to 2C. (b) Cycling performance of the LTO half-cell and (c) Si/graphene – LTO full cell. Both cells were tested under same test conditions as the Si/graphene – SeS_2 full-cell.; the window voltage was 1.0 - 2.85 V at the C-rates from C/10 to 2C.

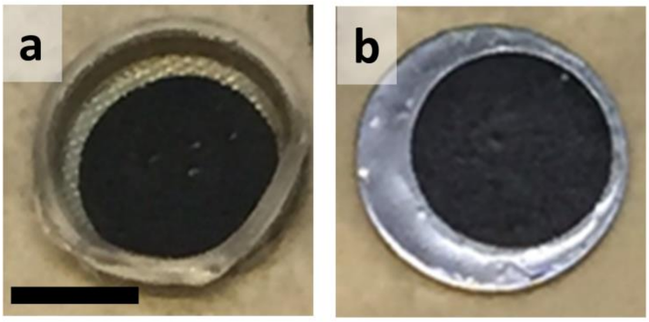

Supplementary Figure 4 │ As-disassembled Si/graphene – SeS_2/CDC coin type full-cell after 1,500 cycles. (a) Si-graphene anode and (b) SeS_2/CDC cathode before DMC washing. Both electrodes and electrolyte were visually similar to their pristine states. Scale bar, 10 mm.

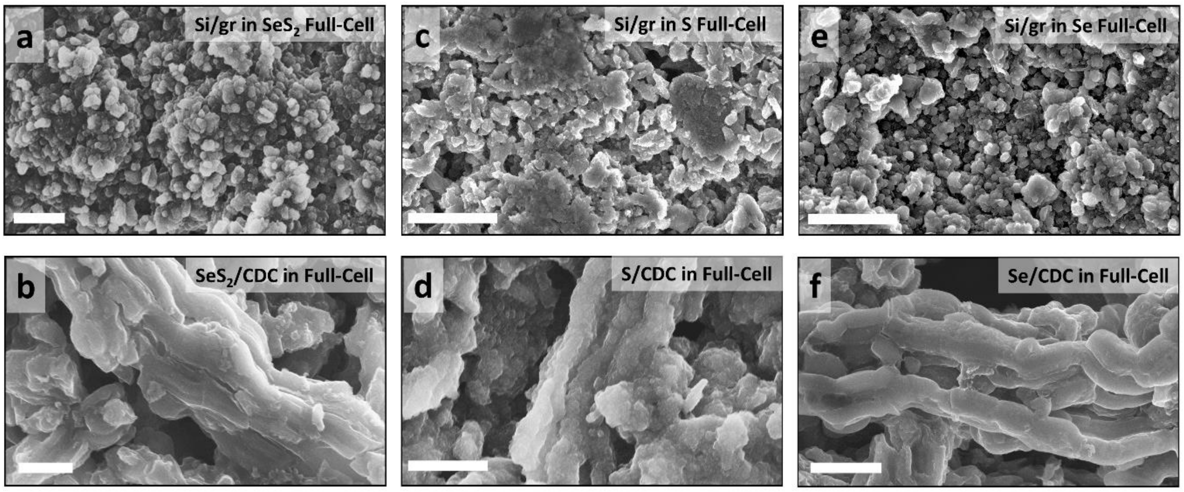

Supplementary Figure 5 │ High resolution-scanning electron microscopy surface images of cycled anodes and cathodes for three full cells. The cycled anodes and cathodes were imaged after 1,000 cycles (a) Si/graphene anode – (b) SeS_2/CDC cathode, (c) Si/graphene anode – (d) S/CDC cathode (a-c), and (e) Si/graphene anode – (f) Se/CDC cathode. When comparing with their pristine (uncycled) morphologies of figure 1a-b, the anode and cathode from the Si/graphene anode – S/CDC full-cell had significant changes; the anode showed many deposits (composed of C, S and O by EDS) on Si particles, and hence the porosity decreased, and the cathode lost crystalline surface of CDC. Scale bars, 10 µm (a, c, e) and 3 µm (b, d, f).

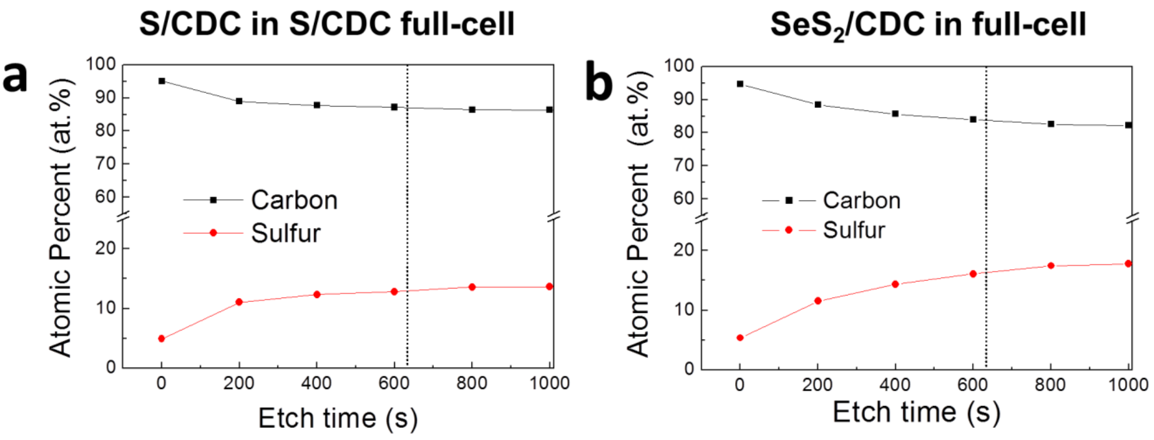

Supplementary Figure 6 │ X-ray photoelectron spectroscopy sulfur 2p analysis of the electrodes in the cycled full-cells. Depth profile (for carbon and sulfur) of the (a) S/CDC and (b) SeS_2/CDC cathode from after 1000 cycles in full cells.

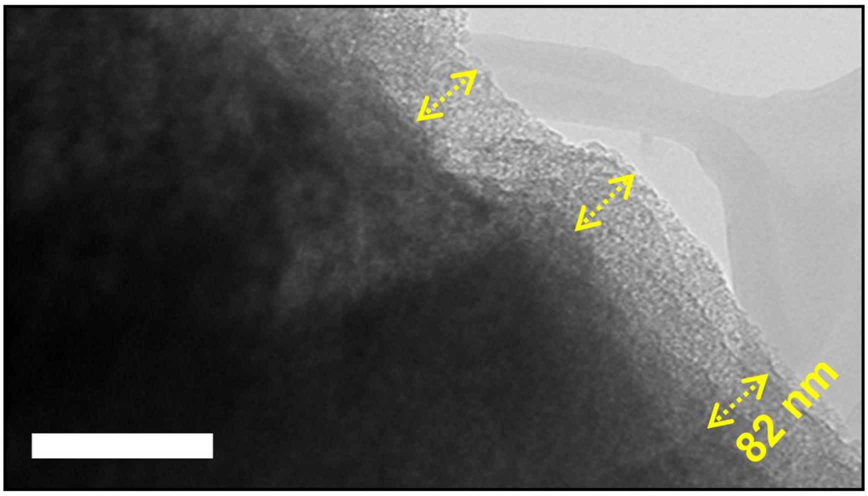

Supplementary Figure 7 | Transmission electron microscope image of the cycled cathode from the SeS_2/CDC full cell. TEM bright-filed image of SeS_2/CDC cathode in the Si/graphene –SeS_2/CDC full-cell after 1000 cycles. Scale bar, 200 nm.

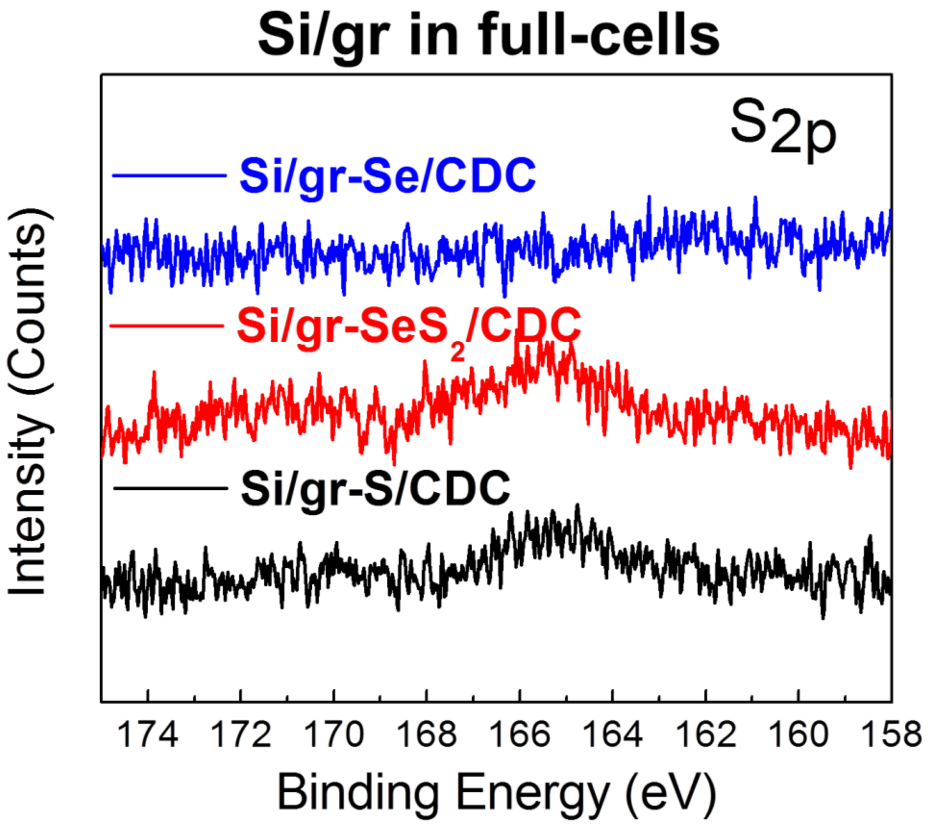

Supplementary Figure 8 | Surface X-ray photoelectron spectroscopy analysis of the cycled Si/graphene anodes in the SeS_2/CDC full cells. XPS surface analysis after 1000 cycles for the Si/graphene anode from the Si/graphene – Se/CDC, Si/graphene – SeS_2/CDC, and Si/graphene – S/CDC full-cells.

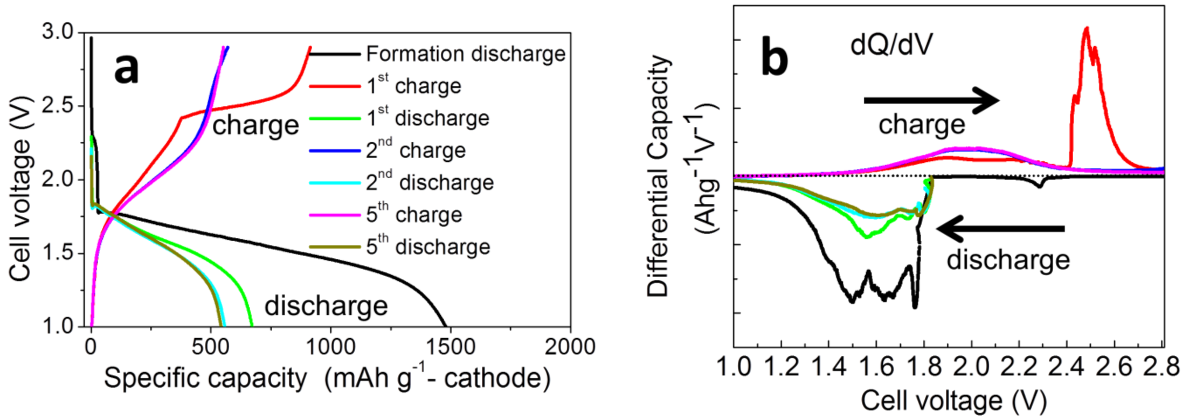

Supplementary Figure 9│ Voltage-capacity (V-C) curves and their corresponding differential capacity curves (dQ/dV) for the Si/graphene – SeS_2/CDC full-cell during the first five cycles. During the formation discharge, various oxidized peaks are detected between 1.5 and 1.75 V due to the formation of $LiS(/Se)_x$ compounds and during the 1st charge, various reduction peaks are detected between 2.4 and 2.6 V due to the SEI formation on anode as reduction product.

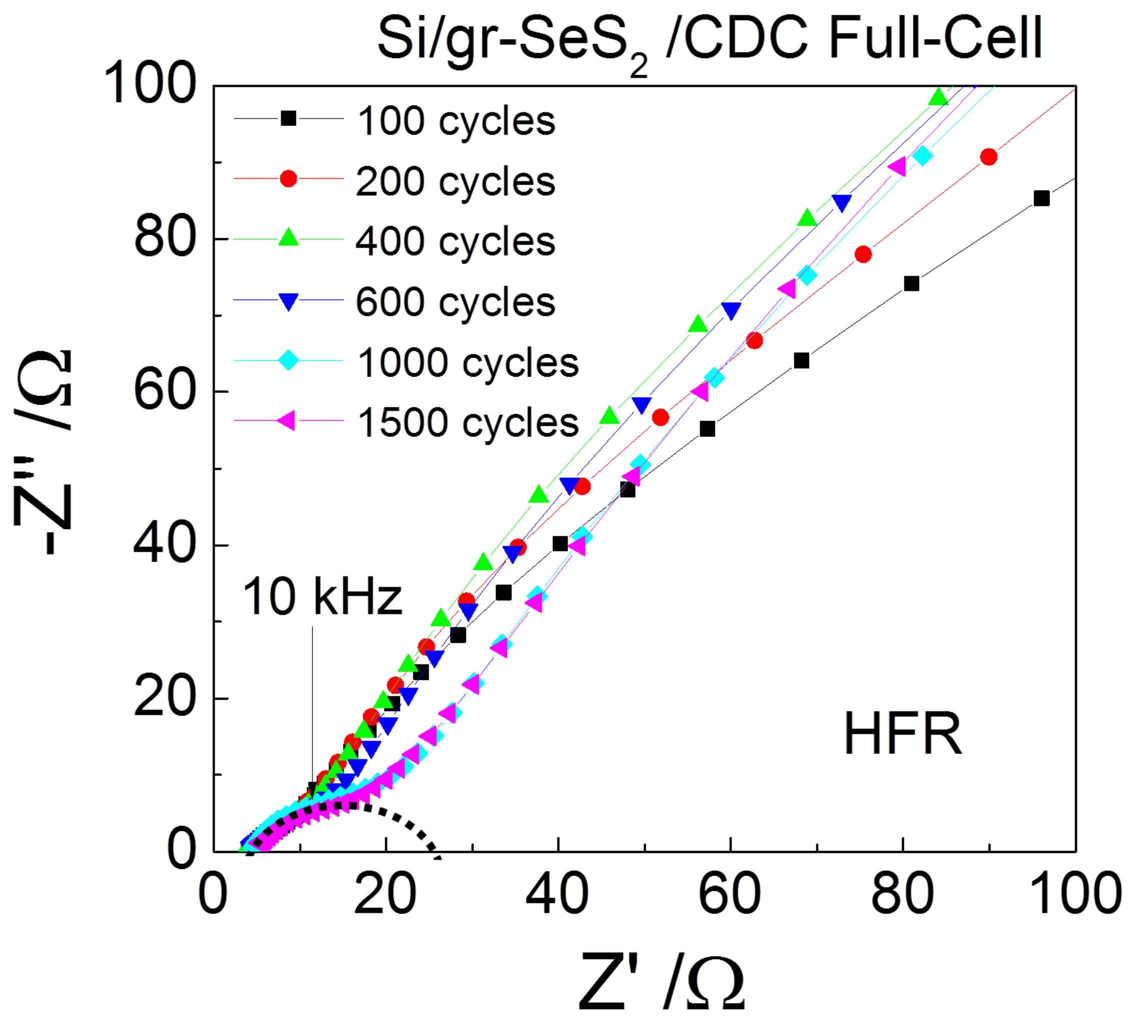

Supplementary Figure 10 | Electrochemical Impedance Spectroscopy of the Si/graphene – SeS_2/CDC full-cell with cycle number. With an increase in cycle number from 100 to 1500, EIS curve in high-frequency resistance (HFR) range remained constant. The HFR curve of EIS is related to the ionic and electrical resistance of SEI film.

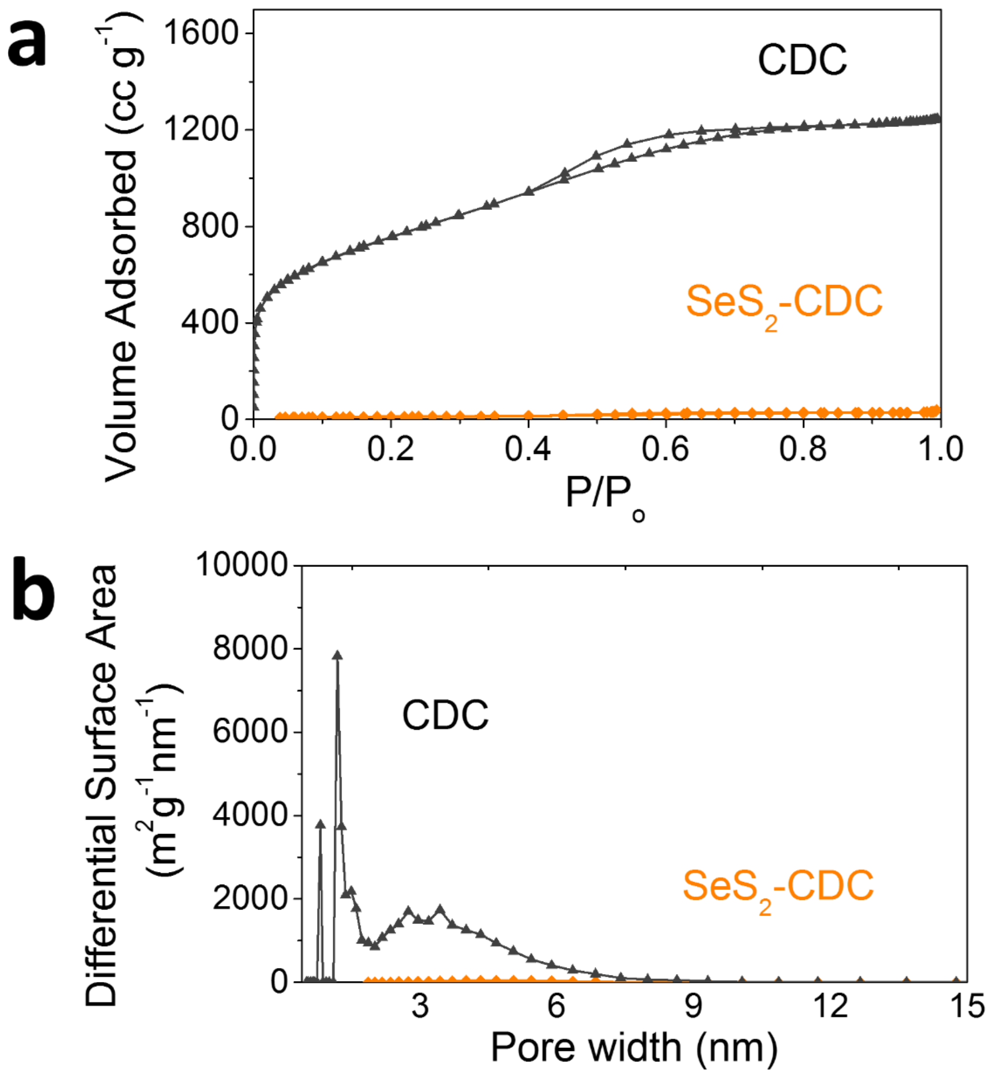

Supplementary Figure 11 | N_2 sorption measurement result and pore distribution of CDC and SeS_2/CDC for cathode. Large CDC specific surface area of ~2740 m^2 g^{-1} and total volume of 1.92 cm^3g^{-1} are attractive for achieving high SeS_2 content in the composites. Pore size distribution acquired by DFT calculation validate that synthesized CDC contain both micropores (< 2 nm) and small (2-7 nm) mesopores. After SeS_2 infiltration, the specific surface and pore volume decreased to ~40.5 m^2/g and ~0.06 cm^3/g respectively, demonstrating that most of pores were successfully filled with SeS_2

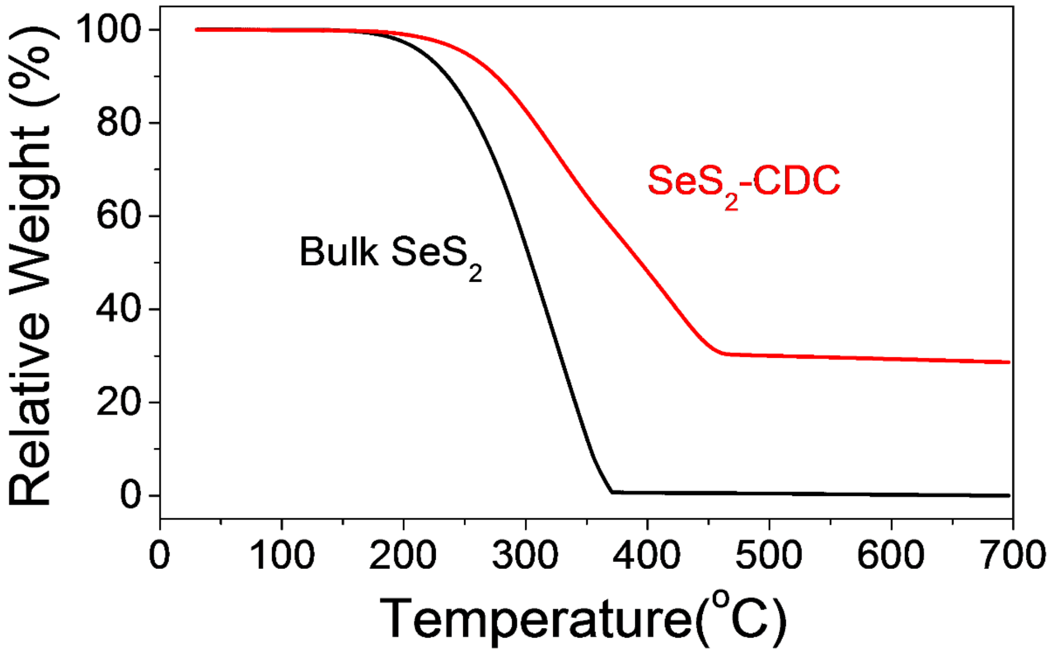

Supplementary Figure 12│ Thermo-Gravimetric Analysis (TGA) curves of Bulk SeS2 and SeS_2/CDC electrode for cathode. The thermal behaviors of the pure SeS_2 and SeS_2/CDC were studied via thermo-gravimetric analysis (TGA) studies under N_2. The weight of both SeS_2/CDC and pure SeS_2 begin to reduce at ~200 °C. Pure SeS_2 was fully evaporated at ~360 °C, but the SeS_2 in SeS_2/CDC was preserved up to ~460°C. These thermal behaviors confirm stronger bonding between SeS_2 and CDC within nano-pores of the SeS_2/CDC.
